# Effects of Oxidized Metal Powders on Pore Defects in Powder-Fed Direct Energy Deposition

**DOI:** 10.3390/mi15020243

**Published:** 2024-02-06

**Authors:** Jong-Youn Son, Ki-Yong Lee, Seung Hwan Lee, Chang-Hwan Choi

**Affiliations:** 1 Department of Mechanical Engineering, Stevens Institute of Technology, Castle Point on Hudson, Hoboken, NJ 07030, USA; json4@stevens.edu; 2 Automotive Materials & Components R&D Group, Korea Institute of Industrial Technology, 9, Cheomdanventure-ro 108beon-gil, Buk-gu, Gwangju 61007, Republic of Korea; kylee@kitech.re.kr; 3 School of Mechanical Engineering, Hanyang University, 222 Wangsimni-ro, SeongDong-Gu, Seoul 04763, Republic of Korea; seunghlee@hanyang.ac.kr

**Keywords:** direct energy deposition, metal powder, oxidization, defect, pore

## Abstract

Laser-based additive manufacturing processes, particularly direct energy deposition (DED), have gained prominence for fabricating complex, functionally graded, or customized parts. DED employs a high-powered heat source to melt metallic powder or wire, enabling precise control of grain structures and the production of high-strength objects. However, common defects, such as a lack of fusion and pores between layers or beads, can compromise the mechanical properties of the printed components. This study focuses on investigating the recurrent causes of pore defects in the powder-fed DED process, with a specific emphasis on the influence of oxidized metal powders. This research explores the impact of intentionally oxidizing metal powders of hot work tool steel H13 by exposing them to regulated humidity and temperature conditions. Scanning electron microscopy images and energy-dispersive X-ray spectroscopy results demonstrate the clumping of powders and the deposition of iron oxides in the oxidized powders at elevated temperatures (70 °C for 72 h). Multi-layered depositions of the oxidized H13 powders on STD61 substrate do not show significant differences in cross sections among specimens, suggesting that oxidation does not visibly form large pores. However, fine pores, detected through CT scanning, are observed in depositions of oxidized powders at higher temperatures. These fine pores, typically less than 250 µm in diameter, are irregularly distributed throughout the deposition, indicating a potential degradation in mechanical properties. The findings highlight the need for careful consideration of oxidation effects in optimizing process parameters for enhanced additive manufacturing quality.

## 1. Introduction

Laser-based additive manufacturing (LBAM) processes can be utilized to fabricate parts or prototypes from the ground up via multiple layered depositions. It allows us to print complex-shaped, functionally graded, or customized parts, which can be utilized in various applications [1]. Direct energy deposition (DED) is one of the widely used LBAM processes. It typically uses a concentrated heat source such as a laser or electron beam with powdered or wired metallic material to build layer-by-layer parts. In general, the DED utilizes a relatively high-powered heat source to form a melting pool atop the surface of a substrate within an inert atmosphere with argon gas, and metal powder or wire is simultaneously injected through the laser beam and into the melt pool [2].

The DED is a complex printing process commonly used to repair or add additional material to existing components. It has a high degree of control over grain structures and can produce high-strength and quality objects [3]. In the DED process, there are common defects, which are usually initiated by a poor laser cladding process. The prime causes for the defects are associated with either undesirable deposition such as inadequate clad bonding and oxide layer, or with inconsistent laser power resulting in inadequate energy density [4]. When multiple layers with overlapped beads are deposited, there can be defects such as a lack of fusion and pores between layers or beads. These defects tend to be increased by the irregular surface finish of the previously deposited layer and by the number of layers [5]. The pores are one of the recurrent issues in the DED process, mostly due to trapped gases or not fully molten powders. The defects such as pores and lack of fusion cause stress concentration and discontinuity of columnar grains in as-deposited materials, and the presence of pores significantly deteriorates the mechanical properties of the multiple layered depositions [6,7]. Furthermore, the stress concentration around defects induces micro-cracks and fatigue failure in the as-deposited materials so that the fatigue life of the materials can be decreased [8,9]. It can also limit the application of as-deposited parts in critical conditions that would require high elongation, toughness, and fatigue resistance [10].

Therefore, many studies investigated pore formation and pore reduction in laser additive alloy by optimizing process parameters. For example, Choi et al. [4] studied the characteristics of a component fabricated through the DED process. Wang et al. [11] investigated the tendency of pore formation by taking account of all possible sources reported in the literature, namely, material and process parameters. Son et al. [12] optimized the process parameters with the response surface methodology (RSM) to quantitatively derive a set of process parameters that can minimize the defects in the powder-fed DED process. They demonstrated how precisely the predicted models approximate the actual experimental results. Ning et al. [13] studied the influences of ultrasonic frequency on melt pool formation, porosity, and thermal-dependent properties. The flow of molten material generally influences to trap gases during deposition and forms pores in as-deposited materials, and some studies investigated an in situ monitoring of the pore formation and analyzed the causes of pores in various ways. Ren et al. [14] developed a methodology for detecting the generation of keyhole pores in laser powder bed fusion (LPBF) by integrating experimental data, multi-physics simulation, and machine learning to discover the distinctive keyhole oscillation behavior associated with keyhole porosity. Yang et al. [15] investigated the process–structure–property correlation including microstructures and pore defects on the LPBF process via X-ray computed tomography and evaluated the damage evolution, failure mechanism, and resulting mechanical properties via in situ tensile tests. Chen et al. [16] proposed a novel in situ defect detection strategy in the DED using a microphone with deep learning. Wang et al. [17] investigated the pore formation mechanisms and pore formation dynamics unique to the spherical-powder delivery in the DED process by using in situ highspeed and operando X-ray imaging. However, there is still a lack of studies on recurrent causes in terms of material conditions such as the degree of oxidation of metal powders on the defects and pore formation. Thus, a more extensive investigation of the causes that commonly affect the defects including the material condition itself is desired.

This study aims to investigate recurrent causes of the formation of defective pores in an experimental environment. In particular, it studied how oxidized metal powders influence the formation of pores in the powder-fed DED process. To simulate the oxidation of metal powders, metal powders are exposed to the environment with regulated humidity and temperature so that the powders are intentionally oxidized. The oxidation of metal powders is examined by scanning electron microscopy (SEM) and energy-dispersive X-ray spectroscopy (EDS). The oxidized powders are then deposited via the powder-fed DED process, and their depositions are examined by SEM and a 3D scanner and compared to the depositions of unoxidized powders under the identical optimized process parameters to investigate exclusively how the oxidized powders affect the formation of defective pores.

## 2. Mechanisms of the Formation of Pores in DED Processes

**Figure 1:**
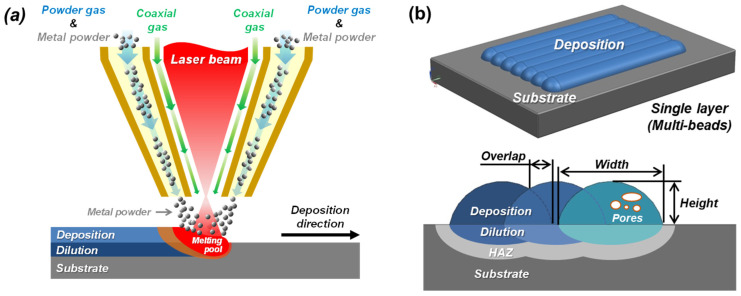
Schematics of (**a**) the powder-fed DED process and (**b**) defective pores formed in the deposition.

**Figure 2:**
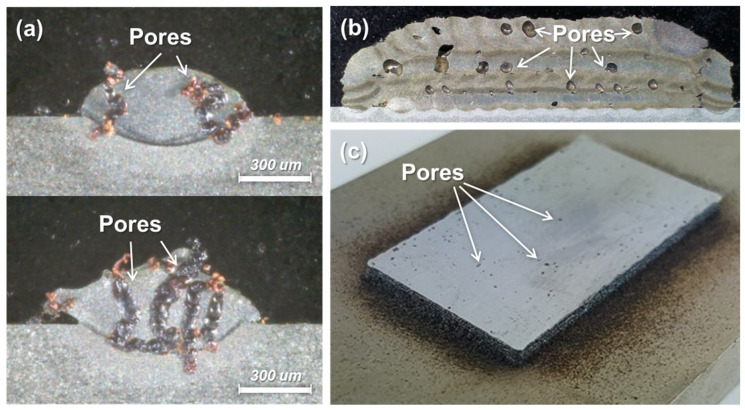
Examples of pores formed in DED processes. (**a**) Pores in single beads. (**b**) Pores in multiple layered deposition. (**c**) Pores on the top surface of deposition.

Figure 1 depicts the schematics of the powder-fed DED process. The DED process utilizes Ar gas for injecting metal powders and directing the metal powders to the melting pool. In the DED process, a laser beam is irradiated to a substrate, and thus a melting pool forms on the substrate. Then, metal powder is simultaneously fed into the melting pool as shown in Figure 1a, and a single bead is deposited onto the substrate with solidification of the molten powders and melting pool. This single bead is overlapped with the previously deposited single bead, and the overlapped single beads become a layer. Then, the deposited multiple layers become a structure. In the DED process, pores naturally occur because of the imperfections in powders as well as multiple layered depositions [18,19]. Evaporated gases from molten powders can be trapped inside the deposited beads or layers and form pores in the deposited metal as illustrated in Figure 1b. Figure 2a,b show examples of actual pores formed in single beads and multiple layers, respectively. Figure 2c shows the porous surface of as-deposited metal.

In general, gas pores may form in the melting pool in the DED processes through four main mechanisms. First, oxygen contained in the melting pool can react with carbon so that CO gas is produced and forms pores [10]. Secondly, the superficial oxide on the metal powder or substrate can be transported into the melting pool so that the oxide can react with the carbon dissolved in the molting pool, resulting in the release of CO gas in the melting pool and, hence, pores. Thirdly, the deposited powders can contain pores within them, and the pores cannot escape during the rapid solidification of molten powders. Then, it can result in the formation of pores in the beads or layers [20]. Lastly, process gases such as shielding gas, powder carrier gas, and coaxial gas can be introduced into the melting pool and cause pores [21]. In this study, we investigate the effects of the oxidation of metal powders on the formation of pores in the powder-fed DED process.

## 3. Materials and Methods

### 3.1. Materials

**Figure 3:**
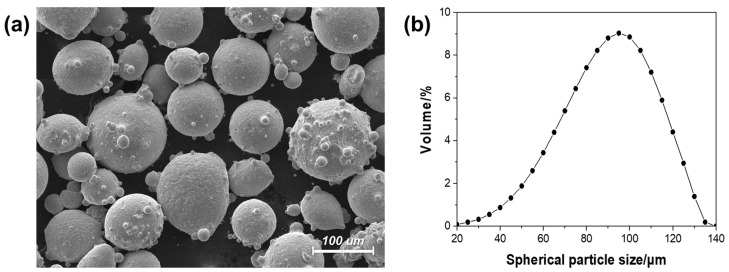
(**a**) Scanning electron microscope (SEM) image of the H13 powders and (**b**) the distribution curve of their particle sizes.

The powder of hot working tool steel (AISI H13, Carpenter, Philadelphia, PA, USA) was used as a metal powder in the powder-fed DED process, and KS STD61 (SeAH CSS, Changwon-si, Republic of Korea) was used as the substrate. Table 1 summarizes the chemical compositions of the H13 and the STD61. The H13 powders consist of spherical particles with diameters of 50–100 μm, as shown in Figure 3.

### 3.2. Oxidation of Metal Powders

**Figure 4:**
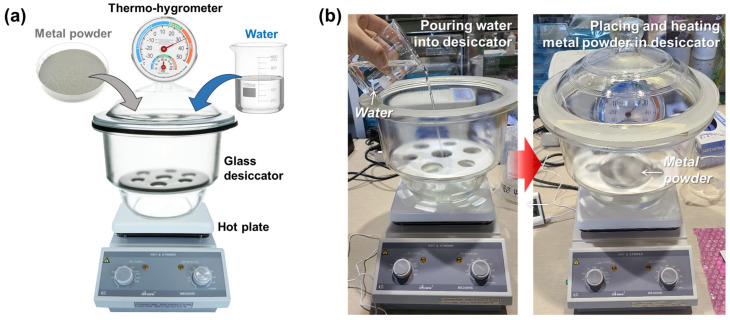
(**a**) Configuration and (**b**) preparation of oxidation setup for metal powders.

The metal powders were made to oxidize in the controlled humidity, temperature, and duration inside the glass desiccator, as shown in Figure 4a. The temperature was controlled by using a hot plate on which the glass desiccator was placed. To expedite the oxidation, the bottom of the glass desiccator was filled with water, which provided the inside with a humidity of 100% RH. When the targeted temperature and humidity were reached in the desiccator, metal powders (60 g) placed in the Petri dish of glass were put over the water, as shown in Figure 4b. The oxidation duration was varied to investigate the effects of the degree of oxidation on the formation of pores in the DED process. Table 2 summarizes the oxidation conditions that were applied for this study.

### 3.3. Characterizations of Metal Powders

After the oxidation of the metal powders, the weight of the oxidized powders was measured by A&D Semi-micro Analytical Balances GR-200 (A&D Co., Ltd., Tokyo, Japan) to examine the change in their mass due to the oxidation. The oxidized powders were also analyzed by using field-emission scanning electron microscopy and EDS (FE-SEM, JSM-7100f with EDS, JEOL Ltd., Tokyo, Japan).

### 3.4. Deposition of Metal Powders

To demonstrate the influence of the oxidation of metal powders on the formation of pores, the oxidized H13 powders were deposited on the STD61 substrate (100 mm × 50 mm × 15 mm) via DED machine using CO_2_ laser (MX3, InssTek, Daejeon, Republic of Korea). Table 3 shows the process parameters of the powder-fed DED. They were optimized with the original (i.e., non-oxidized) metal powders by using response surface methodology (RSM).

### 3.5. Characterizations of Deposited Microstructures

To examine the formation of pores in the deposited microstructures, the deposited beads or layers were partially cross sectioned by using TechCut 5 (Allied High Tech Products Inc., Compton, CA, USA). The cross-sectioned surfaces were polished by using a polisher (MetPrep 3, Allied High Tech Products, Inc., Compton, CA, USA) and then etched by using the nital etchant (nitric acid 5% + ethyl alcohol 95%) for several seconds. The polished and etched surfaces were examined by using an optical microscope (HRM-300, Huvitz, Anyang, Republic of Korea). The depositions were also analyzed by using a CT scan (TVX-IMT300CT, Techvalley, Seongnam, Republic of Korea) to examine the formation of pores for the entire body.

## 4. Results and Discussion

### 4.1. Oxidized Metal Powders

**Figure 5:**
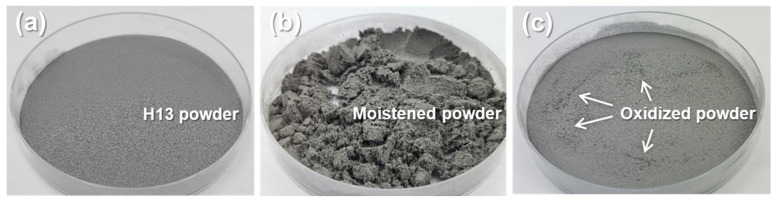
Images of (**a**) the original H13 powders and (**b**,**c**) the oxidized H13 powders at 70 °C for 72 h. The image (**c**) shows the oxidized powders after being dried.

Figure 5a shows the H13 powders before oxidization. After the oxidization of the H13 powders at 70 °C for 72 h in the desiccator, the powders were moistened by humidity as shown in Figure 5b. However, the moistened powders were naturally dried up in minutes, as shown in Figure 5c. The oxidized powders did not show a significant change in their weight. It is attributed to the resolution limit of the employed balance (0.1 mg). The oxidized powders at 30 °C for 12, 24, and 72 h did not apparently show any difference from the original non-oxidized H13 powders. However, the oxidized powder at 70 °C for 24 and 72 h apparently showed coarse particles, as indicated by the arrows in Figure 5c.

**Figure 6:**
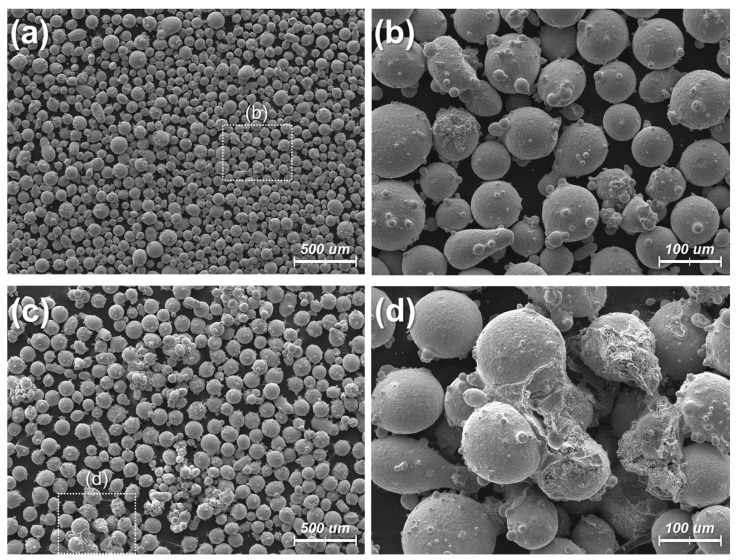
SEM images for the oxidized powders in different conditions: (**a**,**b**) 30 °C for 72 h. (**c**,**d**) 70 °C for 72 h.

Figure 6 shows the SEM images of the oxidized powders at 30 °C for 72 h compared to those at 70 °C for 72 h. As shown in Figure 6a,b, the oxidized powders at 30 °C for 72 h do not show a significant difference from the original non-oxidized powders (Figure 3a). While each powder is mostly covered with smaller particles, the powders remain well dispersed. However, the oxidized powders at 70 °C for 72 h show clumped powders connected by some deposited material, as shown in Figure 6c,d. The SEM images suggest that the size of the individual powers tends to increase with more oxidation, as the oxidation usually increases the volume of the material. However, it was not feasible to statistically analyze the increase in the size because many powders became stuck together after the oxidation.

**Figure 7:**
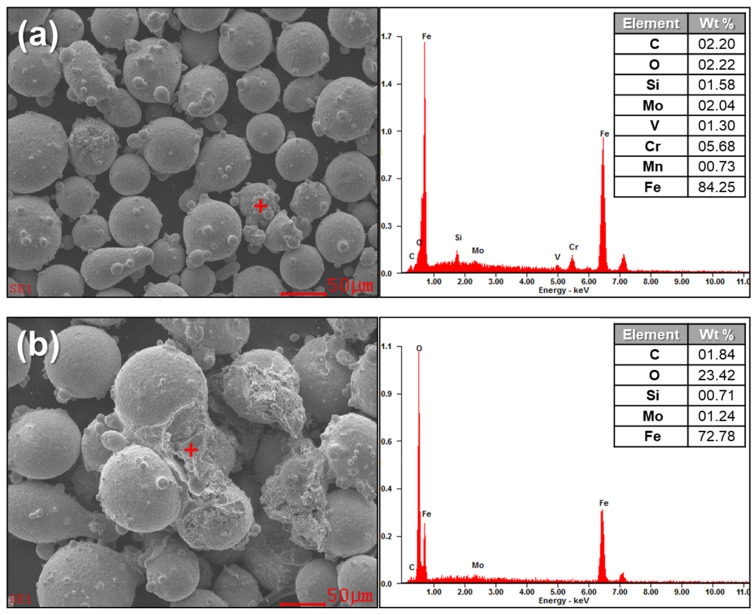
EDS results for the oxidized H13 powders (especially the locations marked with red “+”) in different conditions: (**a**) 30 °C for 72 h. (**b**) 70 °C for 72 h.

Figure 7a,b show the EDS results of the areas where the powders are covered by the smaller particles or the additionally deposited material after the oxidation at 30 and 70 °C for 72 h, respectively. The EDS result of the powders oxidized at 30 °C for 72 h (Figure 7a) shows the typical elements known as main compositions of the H13. It should be noted that the amount of the oxygen element is not as much, as it should be considered as an oxide. In general, the surface of H13 powders has a heterogeneous structure consisting of a thin iron oxide layer with particulate compounds formed by the elements that have high affinity to oxygen [22,23], and the layers are assumed to be irregular parts of the powders or natural oxide layers. In contrast, the EDS result of the powders oxidized at 70 °C for 72 h (Figure 7b) indicates a significantly higher amount of the oxygen element in the deposited area than that found in the irregular parts of the powders oxidized at 30 °C for 72 h. It should also be noted that oxide and iron are two main elements consisting of the deposited area, implying that the deposited material should be iron oxides. The iron oxide in the Fe_3_O_4_ phase is likely to be formed on ferrous alloys under proper temperature and atmosphere [24], and H13 steel can be oxidized greatly in water vapor due to the catalysis of water vapor [22,25]. Thus, the oxide observed on the oxidized powders at 70 °C for 72 h suggests iron oxides.

### 4.2. Deposition of Oxidized Powders

**Figure 8:**
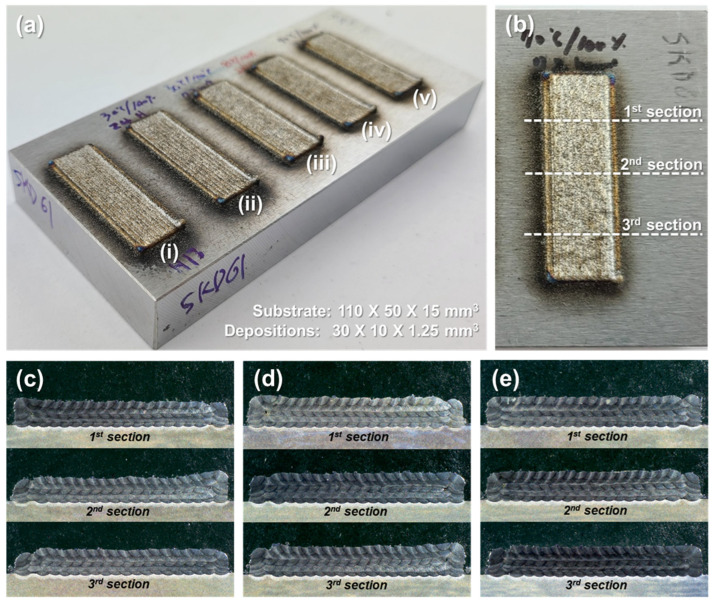
(**a**) Depositions of (i) non-oxidized H13 powders, (ii) oxidized powders at 30 °C for 24 h, (iii) oxidized powders at 30 °C for 72 h, (iv) oxidized powders at 70 °C for 24 h, and (v) oxidized powders at 70 °C for 72 h. (**b**) Locations of the cross-sectioned. (**c**) The cross-section image for the deposition of (i) non-oxidized H13 powders. (**d**) The cross-section image for the deposition of (iii) oxidized powders at 30 °C for 72 h. (**e**) Cross-sectioned image for the deposition of (v) oxidized powders at 70 °C for 72 h.

Figure 8 shows the five-layer depositions of the oxidized H13 powders on STD61 substrate at different conditions, compared to that of the non-oxidized H13 powders, and the cross sections of the depositions. Apparently, the cross-section images do not show significant differences among the specimens, suggesting that the oxidation of the powders would not form relatively large pores that can be visible to bare eyes.

**Figure 9:**
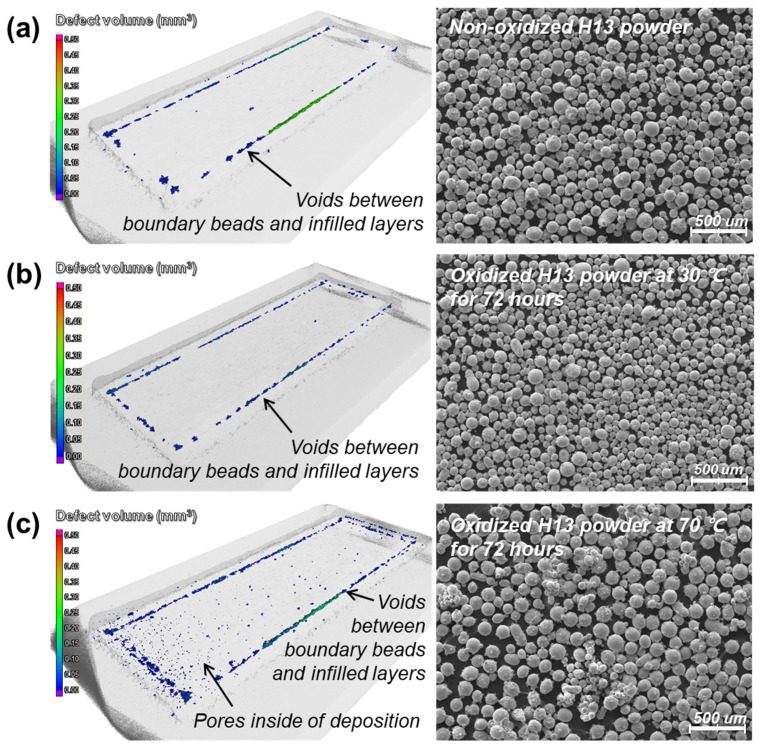
3D scanned results of the depositions of the (**a**) non-oxidized H13 powders, (**b**) oxidized powders at 30 °C for 72 h, and (**c**) oxidized powders at 70 °C for 72 h.

**Figure 10:**
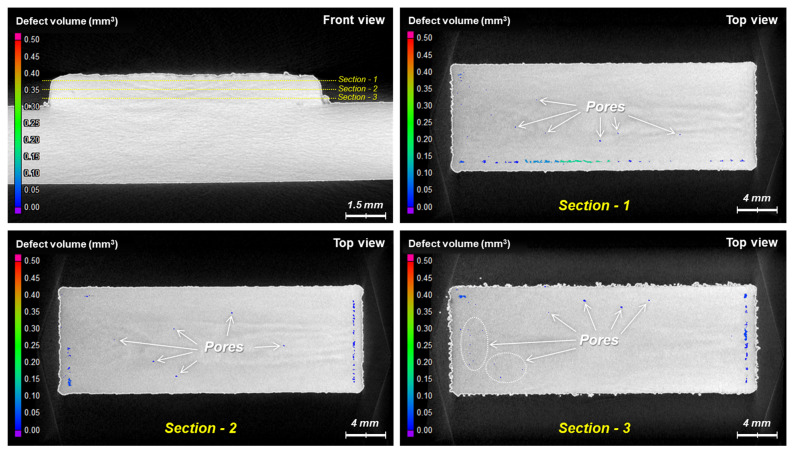
3D scan images of the sectioned layers in the vertical direction of the depositions of the oxidized powders at 70 °C for 72 h.

However, fine pores (typically less than 250 μm in diameter) were detected in CT scanning, as shown in Figure 9 and Figure 10. Figure 9 shows voids between boundary beads and infilled layers for all the specimens, regardless of the oxidation of the powders. These voids were caused by dimensional errors based on the number of inserted beads inside the boundary beads; the slicing program for 3D printing does not allow one to compensate for the remaining space between boundary beads and infilled layers so that such voids appear artificially in the scanned images due to the slicing error. Meanwhile, Figure 9 shows the significant difference in the inner parts of the depositions. In particular, the deposition of the oxidized powders at 70 °C for 72 h (Figure 9c) shows many fine pores irregularly distributed throughout the deposition, whereas the depositions of the non-oxidized powders and the oxidized powders at 30 °C for 72 h (Figure 9a and Figure 9b, respectively) show few pores inside. Figure 10 shows the sectionally scanned images of Figure 9c in the vertical direction. It suggests that fine pores are distributed throughout the deposition.

It was reported that gas pores could form in the molten track during the DED process of oxidized powders due to the low solubility and high buoyancy of the gas in the melt–fluid and that the size of pores would be less than 250 μm in diameter [26]. They usually form near the laser beam and reside adjacent to the molten track surface owing to the Marangoni flow, and the Marangoni flow entrains the gas pores to different locations inside the molten track via centripetal Marangoni convection [26,27]. The pores shown in Figure 9c and Figure 10 are mostly less than 250 μm in diameter. Thus, it is likely that the gases generated by the oxidized powders are entrapped in the melting pool during the DED process and that the gases cannot escape to atmosphere but form the pores within the melting pool.

## 5. Conclusions

This study investigated the effects of the oxidation of the hot work tool steel H13 on the formation of pore defects in the powder-fed DED process. It shows that the exposure of ferrous metal powders to the environment of high temperature and humidity for a relatively long duration can significantly oxidize the powders and, hence, affect the integrity of the deposition in the DED process by producing fine pores (e.g., less than 250 μm in diameter) throughout the deposition. The result suggests that the iron oxides formed on the oxidized metal powders directly affect the formation of gas pores in such a way that the gases generated by the oxides on the powders are entrapped in the melting pool and then the gases cannot escape from but form the pores inside the melt pool. Such pore defects can deteriorate the mechanical properties of as-deposited materials. Thus, it is desirable to minimize the oxidation of metal powders for high-quality deposition in the DED process.

## Figures and Tables

**Table 1:** Chemical compositions of the AISI H13 and KS STD61 (wt.%).

Material	C	Si	Mn	P	S	Mo	Cr	Ni	N	V	O
AISI H13	0.41	1.0	0.45	<0.015	0.02	1.5	5.33	0.25	0.1	1.05	0.1
KS STD61	0.32	0.8	<0.5	<0.03	0.03	1.0	4.5	<0.25	0.03	0.8	-

**Table 2:** Conditions for the oxidation of metal powders.

Powder Weight	Temperature	Humidity	Time
60 g	30, 70 °C	100%	12 h
			24 h
			72 h

**Table 3:** Process parameters of the powder-fed DED process.

Process Parameters	Value
Laser power	800 W
Powder feeding rate	3.3 g/min
Bead spacing	0.52 mm
Laser scanning speed	800 mm/min
Laser beam diameter	1 mm
Powder gas	2.5 L/min
Coaxial gas	8.0 L/min

## Data Availability

Data are contained within the article.
